# Role of Autologous Platelet-Rich Fibrin in Chronic Non-healing Ulcers With Various Etiologies in a Tertiary Care Rehabilitation Centre: A Case Series

**DOI:** 10.7759/cureus.68709

**Published:** 2024-09-05

**Authors:** Arvind K Sharma, Arunpreet Kaur, Satyasheel S Asthana, Ivanah P Nongrum, Siddharth Rai, Kumari Sunaina

**Affiliations:** 1 Physical Medicine and Rehabilitation, All India Institute of Medical Sciences Raebareli, Raebareli, IND; 2 Transfusion Medicine, All India Institute of Medical Sciences Raebareli, Raebareli, IND; 3 Physical Medicine and Rehabilitation, Sanjay Gandhi Postgraduate Institute of Medical Sciences, Lucknow, IND; 4 Endocrine Surgery, Sanjay Gandhi Postgraduate Institute of Medical Sciences, Lucknow, IND

**Keywords:** platelet-rich plasma/prp, guillain-barre syndrome, platelet-rich fibrin, prf, ischaemic ulcer, regenerative medicine, spinal cord injury, non-healing ulcers, chronic ulcers, case series

## Abstract

Introduction

Chronic non-healing ulcers are defined as a discontinuity or break in the integrity of skin that is not healing in a reasonable period of time due to an underlying systemic etiology. Despite using conventional initial treatment and many other available dressing options, such wounds are difficult to completely heal, thus affecting the progress of rehabilitation measures and compromising functional improvement and quality of life.

Materials and methods

In this case series, platelet-rich fibrin (PRF) was applied to eight wounds from six patients. The patients included had various etiologies (including spinal cord injury, peripheral vascular disease, Guillain-Barré syndrome, and diabetic foot ulcer) with chronic non-healing wounds over different anatomical locations on the body. Pressure ulcer scale for healing (PUSH) score, surface area, and volume of the wounds were evaluated and monitored weekly after PRF dressing. We have applied PRF every week. On average, two PRF dressings were applied, the maximum being three applications.

Results

The maximum healing rate in terms of PUSH score was observed to be 3.84% per day, and the minimum was 1.19% per day. The maximum healing rate in terms of surface area was observed to be 5.89% per day, and the minimum was 1.78% per day. Three of the wounds showed complete closure. The maximum follow-up period was 10 weeks. The percentage mean Functional Independence Measure (FIM) improvement was calculated to be 15.87% ± 14.04 during the course of hospitalization after PRF application.

Conclusion

Based on the results, we can conclude that PRF showed accelerated improvement in the healing of chronic non-healing ulcers of various etiologies at different anatomical locations. It has proven to be a safe and effective method, thereby improving their quality of life and functional independence in performing activities of daily living. To our knowledge till date, no other study in a rehabilitation setting has been done on patients having non-healing ulcers due to various etiologies and at different anatomical locations.

## Introduction

Chronic non-healing ulcers are defined as a discontinuity or break in the integrity of skin that is not healing in a reasonable period of time due to an underlying systemic etiology despite using conventional initial treatment [1]. The normal process of wound healing includes the steps of hemostasis, inflammation, proliferation, and remodeling [2]. When any of these stages get affected, it disrupts the entire wound-healing process and results in non-healing ulcers. They may be of variable etiology, such as pressure injuries (PIs) and diabetic and vascular ulcers, where the epithelium disintegrates due to pressure, ischemia, infection, and metabolic causes [3].

The ultimate goal in the treatment of such chronic ulcers is to enable closure as quickly as possible. There are a number of methods used to optimize the wound, which include daily cleaning and dressing of the wound, prescription of appropriate antibiotics according to culture and sensitivity reports when required, surgical debridement, application of negative pressure wound therapy (NPWT) or vacuum-assisted closure (VAC), control of blood sugar levels, adequate nutrition, and treatment of systemic causes contributing to the ulcer formation and mechanical off-loading [4]. A number of factors leading to non-healing ulcers include external factors like repeated trauma, pressure, shearing stress, moisture, poor body hygiene, behavioral factors like smoking and alcohol consumption, poor cognitive function, internal factors like sensory loss, fever, anemia, and malnutrition, increasing age, systemic illnesses, immune suppression, bacterial/viral/fungal infections, malignancies, and comorbidities including vasculitis and coagulation disorders [5].

As of now, the current modalities used to treat non-healing ulcers include patient and caregiver education, frequent change of posture to redistribute the weight in order to avoid the formation of PIs, ensuring proper nutrition, avoiding smoking and alcohol consumption so as to reduce risk factors contributing to the chronic nature of the ulcers as discussed above [6]. In general, stage 1 and 2 PIs are usually treated with local care non-surgically by conventional dressing with 0.9% normal saline under aseptic conditions. Stage 3 and 4 PIs often remain as chronic non-healing ulcers and may require surgical intervention. A positive wound culture report does not necessarily act as an indication for administering systemic antibiotics unless the patient shows signs and symptoms of systemic sepsis, spreading cellulitis, and underlying osteomyelitis [7]. The wound can be optimized by means of debridement, which can be done surgically, to remove regions of eschar and thick necrotic tissue as it prevents wound healing, harbors bacteria, and obscures the deep regions of the pressure ulcer. Debridement of PIs can be done using different approaches, such as autolytic, enzymatic, mechanical, and surgical debridement [8].

Autologous platelet-rich plasma (PRP) therapy is a first-generation method of using the patient’s peripheral blood sample to produce a platelet-rich concentrate that releases growth factors aiding in tissue regeneration. These include platelet-derived growth factor, transforming growth factor-beta, vascular endothelial growth factor, fibroblast growth factor, and epidermal growth factor. PRP is more beneficial when compared to whole blood because it has a mononuclear cell content, enabling the acceleration of the healing process in contrast to the acute anti-inflammatory cell profile of whole blood [9]. It has been used as an intervention for non-healing ulcers due to its regenerative properties. However, there are certain disadvantages that can be overcome by using autologous platelet-rich fibrin (PRF). PRP has been popularly used for the treatment of PIs. However, it has been noticed that, with time, the use of anticoagulants delays the process of wound healing.

PRF therapy is a second-generation method where anticoagulants are not used for preparation [10]. In addition to all the growth factors that PRP has, PRF also has an abundance of neutrophils, which additionally prevent infections at the site of the PI [11]. Human mesenchymal stem cells are also present in the scaffold of PRF, which accelerates tissue regeneration [12]. Another benefit is that it does not require the addition of anticoagulants and other additives; hence, it preserves the properties in its natural state [13]. PRF shows extended release and prevention of the proteolytic breakdown of growth factors [14]. PRF has also been seen to improve the rate of healing of wounds at the cellular level in terms of proliferation, migration, adhesion, and tissue regeneration in a recent systematic review [15].

## Materials and methods

Inclusion and exclusion criteria

In this case series, eight wounds of six patients were included, in which PRF was applied. The patients included were in the age group of 18-75 years with chronic non-healing ulcers with variable diagnoses (including spinal cord injury, peripheral vascular disease, Guillain-Barré syndrome, and diabetic foot ulcer) over different regions of the body. Patients who were hemodynamically stable, had no fever, and had a hemoglobin of more than 12 g/dL and platelet count of more than 1.5 lakh/mL of blood were included.

Patients with coagulation disorders, history of malignancy, pregnant or lactating mothers, and patients with uncontrolled diabetes mellitus were excluded. All patients had been admitted to the Department of Physical Medicine and Rehabilitation at All India Institute of Medical Sciences, Raebareli, India, where patient information, complete physical examination, and laboratory investigations were done. Informed and written consent was obtained prior to the administration of PRF. The nutritional status of all patients was evaluated, serum albumin and protein levels were checked, and a high-protein diet was provided to them.

Management procedure

**Figure 1:**
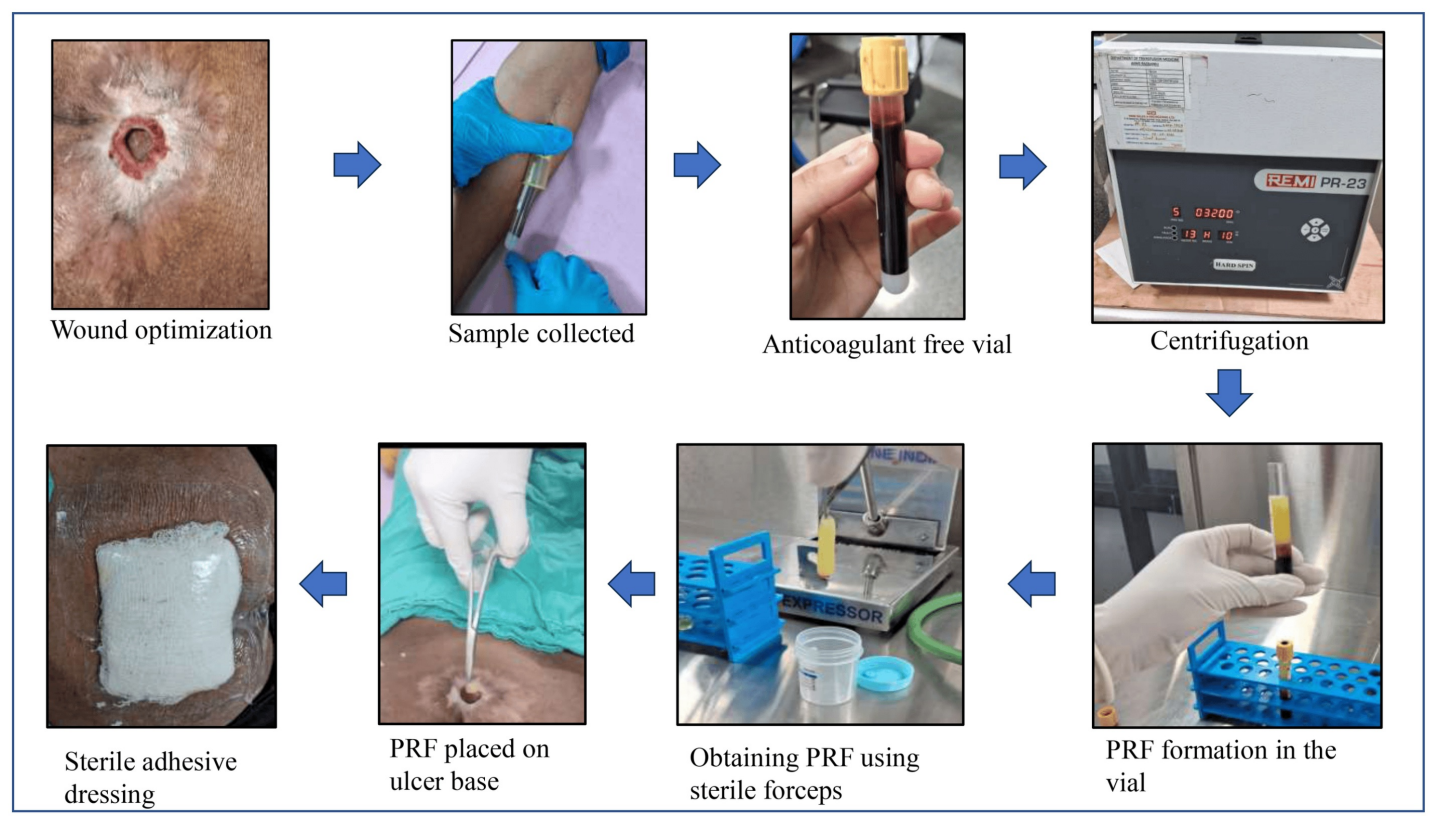
PRF preparation procedure PRF, platelet-rich fibrin

After optimizing the wound by conventional dressing and surgical debridement, the patient was prepared for PRF application. A wound pus culture was done, and appropriate antibiotics, as per sensitivity reports, were administered to the patient as and when required. For preparation of PRF, the patient was taken to the Department of Transfusion Medicine and Blood Bank, where a 10 mL of venous sample was drawn from the patient’s antecubital vein into a sterile vial without any anticoagulant [10]. It was immediately placed in a pre-programmed centrifuge. This was done using a tabletop centrifuge (PR-23) at 3200 rpm for 10 minutes [15]. The plasma was drained, and fibrin was separated, which was placed directly over the base of the ulcer and was covered by a sterile adhesive dressing. It was left in situ for seven days [2]. The dressing was monitored for soakage or leakage, during which it was redone when needed. Figure 1 depicts the image form of the preparation procedure.

Vitals of the patients and dressing/wound were monitored for any adverse reactions, including fever or worsening of the wound.

Hygiene around the wound and perineal hygiene were maintained and monitored. Skin and foot care were done to keep the skin clean and dry. Pressure relief every 15 to 30 minutes was advised during wheelchair sitting, and two hourly changes of posture were done when the patient was on the bed. Pressure-distributing mattresses and cushions were used, and clothes and linen were kept wrinkle-free to reduce shear stress. The maximum duration for which the PRF dressing was kept was seven days in a single sitting. Digital images of the wounds were taken and recorded at baseline, before and after every PRF application. All patients were given individualized rehabilitation programs as per clinical assessment.

The effect of autologous PRF in the healing of chronic non-healing ulcers was evaluated by assessing the pressure ulcer scale for healing (PUSH) score, reduction of size in terms of surface area, and volume improvement wherever feasible. A simple ruler method (length × breadth) was used to measure the surface area, where the length and breadth of the wound were measured in centimeters and multiplied to obtain the surface area in square centimeters. The volume of the PI was measured by filling sterile normal saline from a graduated syringe without overflowing the saline from the brim of the ulcer. PUSH score is evaluated based on surface area, exudate amount, and tissue type, with a score ranging from 0 to 17 [16].

Functional assessment was done using the Functional Independence Measure (FIM) scale. FIM is a measure of function in self-care, sphincter control, transfers, locomotion, communication, and social cognition. The maximum score is 126, which indicates complete independence, and the minimum score is 18, which indicates total assistance. FIM improvement during the course of rehabilitation indicates progress of recovery in functional independence [17].

Healing rate analysis was done in terms of PUSH score, surface area, and volume of the wounds. This was calculated using the formula [18]:

Healing rate percentage improvement in PUSH score (%per day)

= [PUSH score 0 − PUSH score T)]/PUSH score 0 × 100% divided by T

Healing rate percentage improvement in surface area (%per day)

= [surface area 0 − surface area T)]/surface area 0 × 100% divided by T

Healing rate percentage improvement in volume (%per day)

= [volume 0 − volume T)]/volume 0 × 100% divided by T

T = number of days after the first PRF application

PUSH = pressure ulcer scale for healing

PUSH score 0 = initial PUSH score

PUSH score T = PUSH score at T time

Surface area 0 = initial surface area (cm^2^)

Surface area T = surface area at T time (cm^2^)

Volume 0 = initial volume (mL)

Volume T = volume at T time (mL)

Data analysis

Data entry and analysis were done using Microsoft Excel 365, version 2407. Continuous variable is presented as mean and standard deviation, and non-continuous variables are presented as percentages.

## Results

Eight non-healing wounds of six cases, each having one or two wounds of varying etiology, were included and treated with autologous PRF. Among the included patients, four (66.67%) were male and two (33.33%) were female, with a mean age of 35.33 ± 14.65 years. Of the six cases, three (50%) were spinal cord injury patients, one (16.67%) was an ischemic ulcer due to peripheral vascular disease, one (16.67%) was PI in a patient with Guillain-Barré syndrome, and one (16.67%) was a diabetic foot ulcer. Of eight wounds, wounds A and E were grade IV, wounds C and F were grade III, and wounds D and G were grade II PIs according to the National Pressure Injury Advisory Panel (NPIAP) staging of PIs [19]. Wound H was grade 2 according to Wagner’s classification of diabetic foot ulcer [20]. FIM score baseline values ranged from 48 to 121, with a mean of 87.33 and a standard deviation of 27.14. FIM score at the last follow-up ranged from 62 to 123, with a mean of 99.33 and a standard deviation of 26.24. The percentage mean FIM improvement was calculated to be 15.87% ± 14.04.

The details, including demography, diagnosis, location of wound, and FIM score, are depicted in Table 1.

**Table 1:** Demographic details of patients with improvement of FIM score FIM, Functional Independence Measure

Patient	Wound	Age (years)	Gender	FIM at admission	FIM at last follow-up	Diagnosis	Location of wound
1	A	38	Male	86	118	Spinal cord injury	Sacral
2	B	55	Male	121	123	Peripheral vascular disease	Posterior aspect of the right leg
3	C	25	Male	48	62	Spinal cord injury	Sacral
	D						Left greater trochanter
4	E	23	Male	65	72	Guillain-Barré syndrome	Sacral
	F						Right greater trochanter
5	G	21	Female	96	102	Spinal cord injury	Right gluteal region
6	H	50	Female	108	119	Diabetic foot ulcer	Over right heel

The healing rate calculated at the time of discharge in terms of percentage improvement per day has been shown in Table 2.

**Table 2:** Healing rate in percentage per day of PUSH score, surface area, and volume of the wounds of the case series (volume of wounds B, C, D, F, and G were not measurable) PUSH, pressure ulcer scale for healing "-" indicates that the volumes for wounds B, C, D, F, and G were not measurable.

Wound	Healing rate; percentage improvement in PUSH score (%per day)	Healing rate; percentage improvement in surface area (%per day)	Healing rate; percentage improvement in volume (%per day)
A	1.46	3.09	3.33
B	1.19	2.67	-
C	2.38	2.38	-
D	3.84	5.89	-
E	2.04	2.94	4.76
F	1.78	3.43	-
G	2.85	1.78	-
H	2.59	3.57	2.44

The mean with a standard deviation of healing rate per day for PUSH, surface area, and volume of the wounds is depicted in Table 3.

**Table 3:** Mean with standard deviation of healing rate (percentage per day) for PUSH, surface area, and volume PUSH, pressure ulcer scale for healing

Healing rate (percentage per day)	Mean with standard deviation
PUSH score	2.27 ± 0.85
Surface area	3.17 ± 1.22
Volume	3.51 ± 1.16

**Figure 2:**
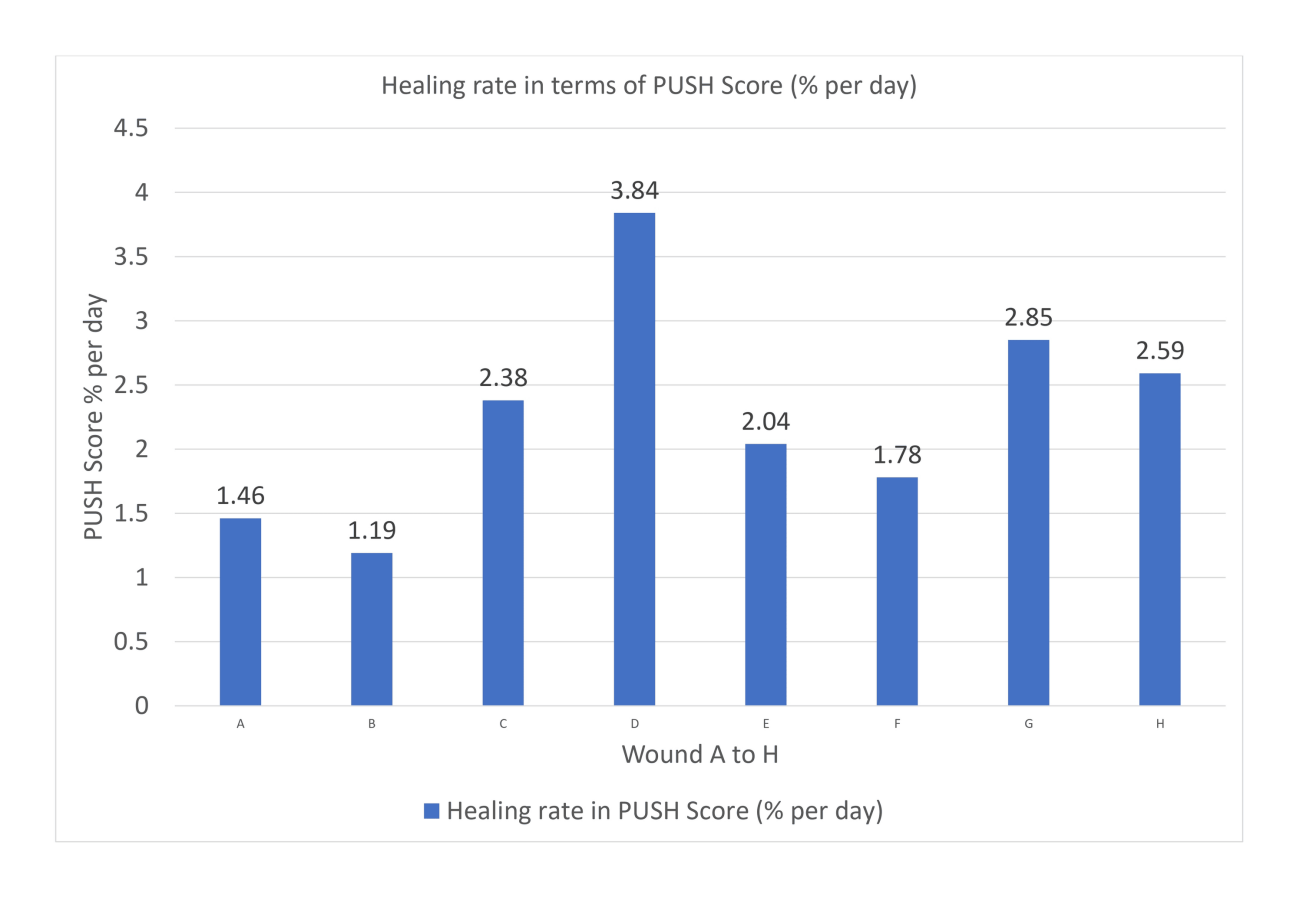
Healing rate of all eight wounds in terms of percentage per day for PUSH score PUSH, pressure ulcer scale for healing

The healing rate in terms of percentage per day for the PUSH score of all eight wounds has been depicted in Figure 2.

**Figure 3:**
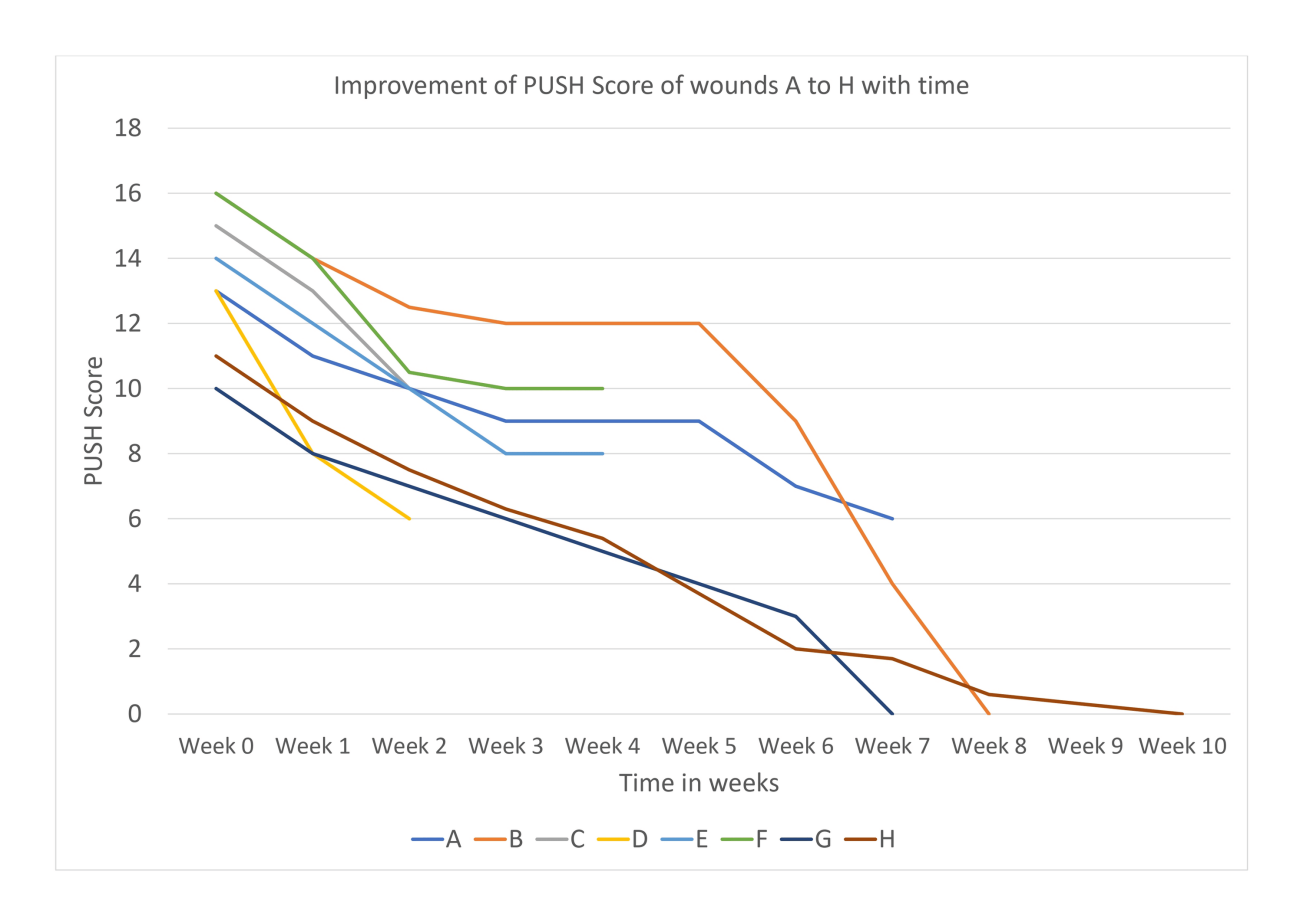
Graphical representation of improvement of PUSH score with time PUSH, pressure ulcer scale for healing

Within the first three weeks of the first PRF application, granulation tissue reappeared over all the wounds, and a considerable reduction in wound size and increase in tissue mass were observed as a sign of healing and improvement. Improvement of PUSH score with time has been depicted in Figure 3.

**Figure 4:**
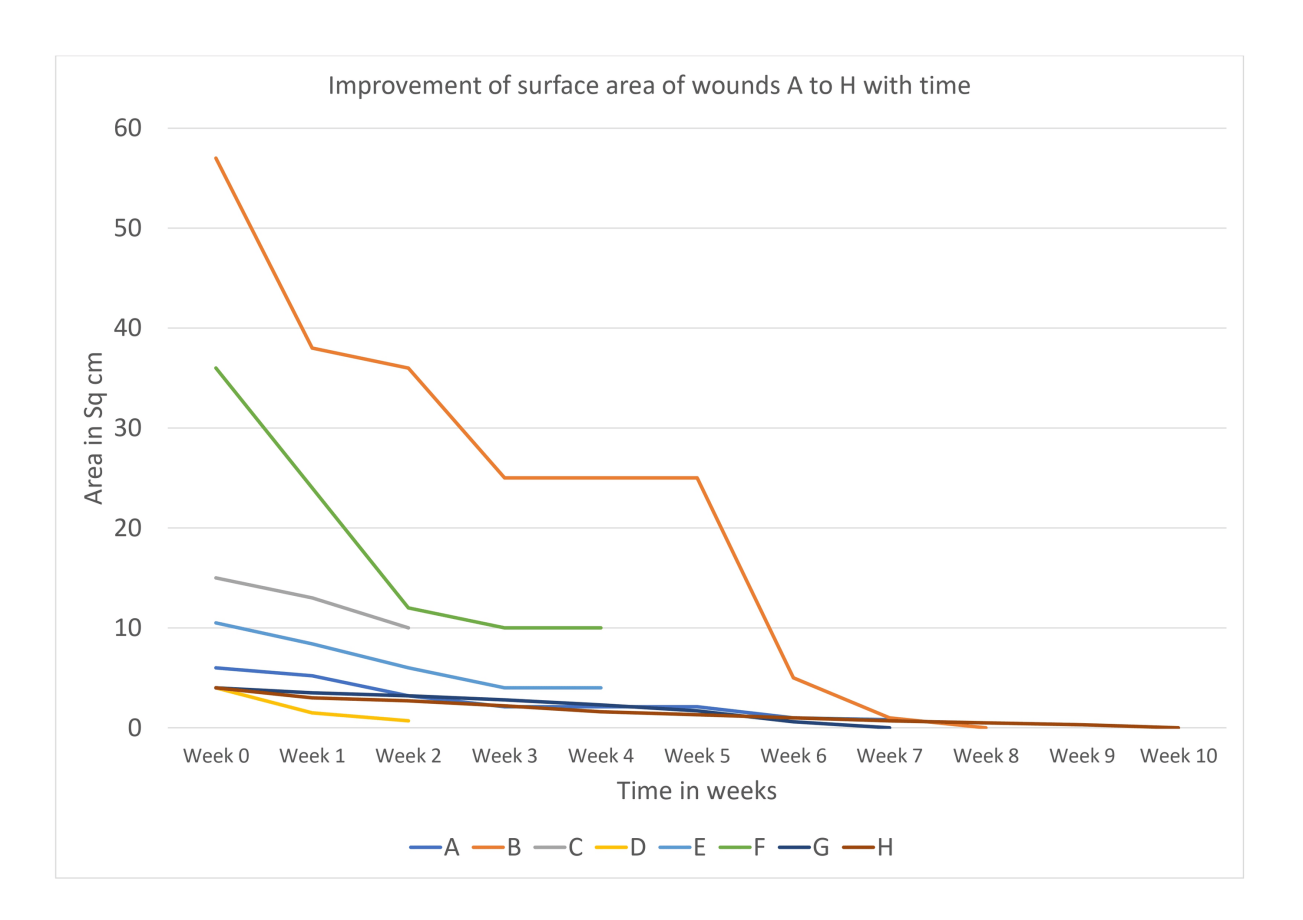
Graphical representation of surface area with time

Improvement of the surface area with time has been depicted in Figure 4.

**Figure 5:**
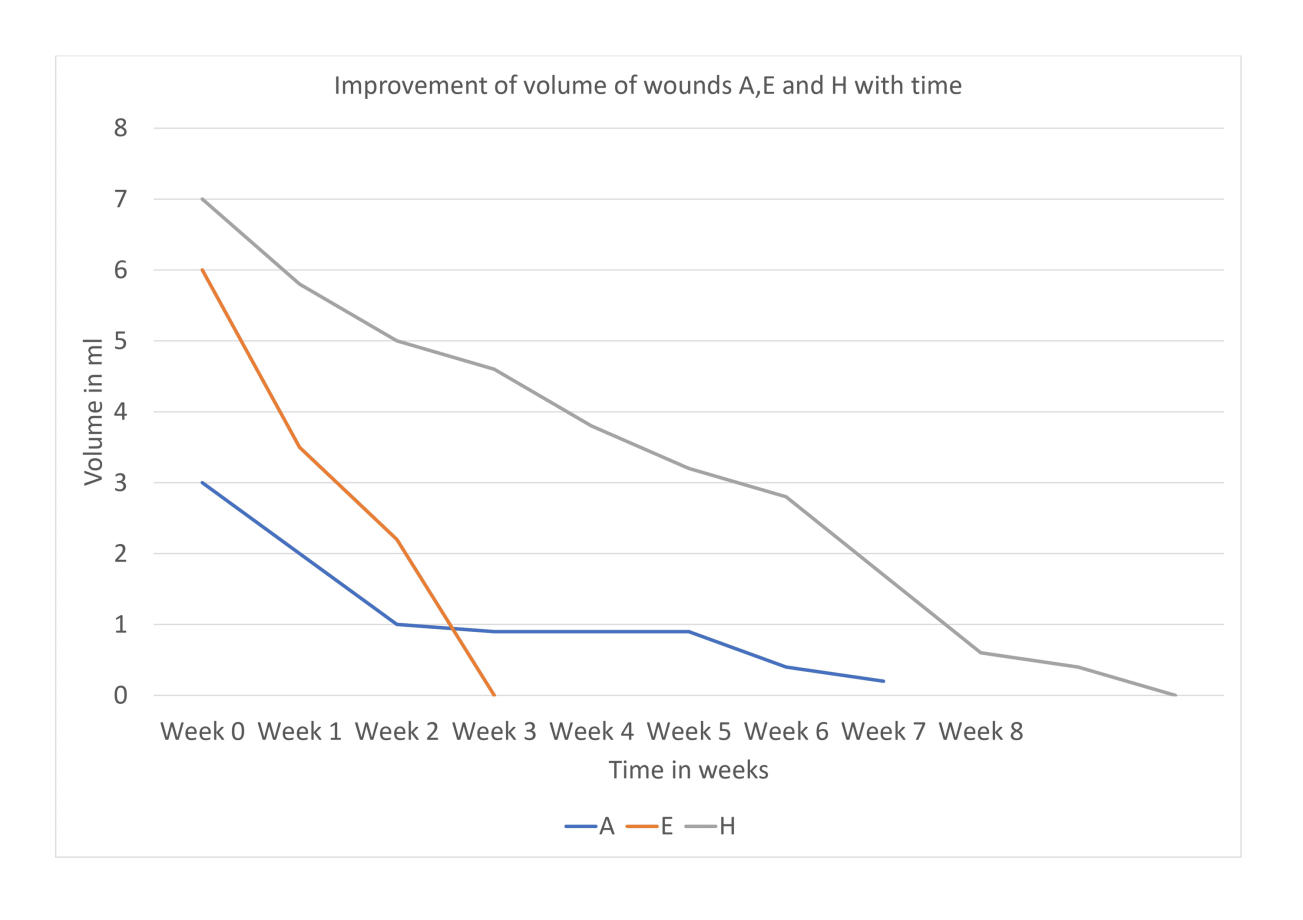
Improvement of the volume of wounds A, E, and H with time

Improvement of the volume of wounds A, E, and H has been depicted in Figure 5.

**Figure 6:**
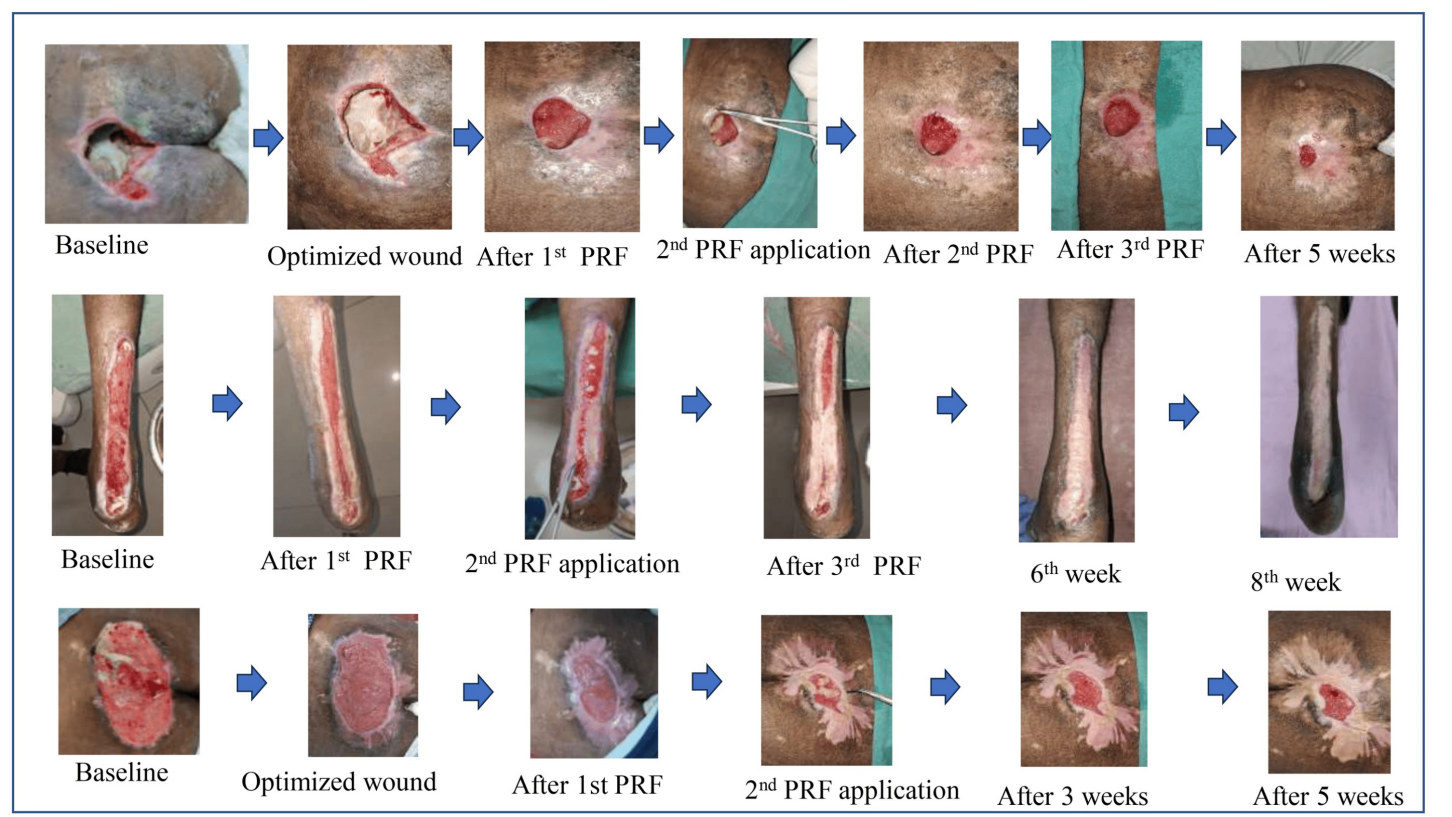
Improvement seen in the wounds indicating the efficacy of PRF application Figure depicting wounds at the time of admission, at the start of PRF application after wound optimization, after serial PRF application, and on the follow-up. PRF, platelet-rich fibrin

Figure 6 shows the wound pictures pre- and post-PRF application, clearly depicting the reduction in wound size with time following PRF administration.

## Discussion

Non-healing ulcers pose a major challenge in developing countries like India and are a burden for patients and healthcare providers [4]. They represent the healthcare, social, and economic burden that affects patient rehabilitation programs and functional recovery. Management of PIs is expensive and requires a holistic team approach. By the term “holistic,” we mean that our focus was not merely on wound management, but it was on the rehabilitation of the patient as a whole, and we should work on the patient’s level of functioning as a dynamic interaction between his/her health conditions, environmental factors, and personal factors. The approach should cover internal, external, psychological, and social aspects of the patient [8]. The primary goal in the non-surgical management of PIs is to optimize the healing conditions and minimize the complications due to long periods of immobilization. Conventional methods such as dressing and debridement of the wound often remain unsuccessful in such wounds. PRF is a new second generation of platelet concentrate that can be used to promote wound healing, bone regeneration, graft stabilization, wound sealing, and hemostasis [12]. The growth factors released from PRF enable mesenchymal cell recruitment during the healing process. Timely closure of PIs aids in avoiding infections, thereby improving patients’ functional recovery and quality of life. It is well known that regenerative measures like the use of blood components can stimulate and accelerate wound healing.

A previous case report by Swarnakar et al. (2022) showed that the application of autologous PRF over a PI had resulted in faster healing. This was used as a novel method in a spinal cord injury patient to treat the PI over the sacral region. There have been no other studies showing the use of PRF in PIs over other anatomical locations in spinal cord injury patients [10]. There are no clear guidelines to define the chronicity of non-healing wounds; however, a range of four weeks to three months has been used in the literature [21]. The duration for which the wound persisted in our case series, prior to the application of PRF, was found to be ranging from six weeks to four months. According to the Wound Healing Society, chronic wounds can be classified into four major categories: pressure ulcers, diabetic foot ulcers, venous ulcers, and arterial insufficiency ulcers [21]. In this case series, PRF has been applied over non-healing wounds of varying etiologies, including diabetic foot ulcer, ischemic ulcer in peripheral vascular disease, PIs in Guillain-Barré syndrome, and spinal cord injury over various anatomical locations of the body. Thus, in our case series, we have three out of four major categories of chronic wounds as classified by the Wound Healing Society. All eight wounds showed significant improvement during and in between the application of the autologous PRF dressing.

Grade 3 and 4 PIs take a toll on the patient and caregivers as they take months to years for recovery, thus acting as a burden to their activities of daily living and also hindering their neuromuscular rehabilitation progress. PRF has been seen to improve the healing rate of such non-healing wounds in a shorter span of time [10]. PRP has been widely used as a therapeutic tool to expedite the healing of chronic ulcers; however, it requires the use of an anticoagulant and also requires a higher quantity of blood to be drawn from the patient. For PRF, there is no need for any other additive, and preparation is easy, less time-consuming, and cost-effective. Its effect remains for a longer period due to the slow release of growth factors over a period of seven or more days [15].

In 2021, Singh et al. showed the use of PRP for the treatment of PIs, where the healing rate in terms of reduction of surface area per day and improvement of PUSH score per day was calculated. Swarnakar et al.’s case report on PRF showed a PUSH score change from 12 to 0 in a time period of four weeks [10], thus giving a healing rate of 3.57% per day in terms of PUSH score. Singh et al. showed an improvement in the PUSH score of 43.14% in six weeks [22], thus giving an improvement percentage of 1.02% per day. In our case series, the maximum healing rate calculated at the time of discharge was found to be 3.86% per day in terms of PUSH score (in wound D), and the minimum was 1.19% per day (wound B). The maximum healing rate for surface area in terms of percentage per day was found to be 5.89% (wound D), and the minimum was 1.78% per day (wound G).

On average, each of the wounds in our study was treated with PRF dressing twice, with a maximum of three applications. The patients showing less than 50% improvement in terms of reduction of surface area within the first two weeks of PRF application were administered the third PRF dressing [23]. The patients were discharged at an average of three weeks from the first application of PRF and were followed up subsequently up to a maximum of 10 weeks. All wounds showed considerable improvement when the patients were discharged and at follow-up. Three of the wounds showed complete healing on follow-up (wound B at week 8, wound G at week 7, and wound H at week 10). None of them developed any adverse reactions such as itching, burning, fever, worsening of the wound, or infection.

All patients were given individualized rehabilitation programs such as patient and caregiver education about disease condition and prognosis, postural care like pressure relief every 15-30 minutes during wheelchair sitting and two hourly changes of posture in bed, moisture control, prevention of contamination of the wound, perineal hygiene, bowel program, mechanical offloading of the wound area, therapeutic exercises, and measures to manage spasticity. For the patient having peripheral vascular disease, an expert opinion was taken from the department of cardiothoracic vascular surgery, and an arteriovenous Doppler of the bilateral lower limb was done. The patient was started on antiplatelet drugs, along with statins and beta blockers. Additionally, ankle range of motion exercises, along with ankle foot orthosis, were given to prevent ankle contracture as part of standard rehabilitation protocol. For the patient with the diabetic foot ulcer, an expert opinion was taken from the Department of General Medicine regarding glycemic control. A total contact cast was applied to offload the wound area. After healing of the wound, customized footwear, therapeutic exercises, and daily self-examination of feet were advised as part of standard rehabilitation protocol. 

Limitations in our study were a low number of subjects due to hemodynamic instability, sepsis, non-acceptance, severe anemia, and poor nutrition. Non-homogeneity in terms of age, etiology, and anatomical location was also a limiting factor. Additionally, there was no control group to compare the healing rate.

## Conclusions

Based on the results from our case series, we can conclude that PRF showed accelerated improvement in the healing of chronic non-healing ulcers of variable etiologies at different anatomical locations. It has proven to be a safe and effective method, thereby improving their quality of life and functional independence in performing daily activities. PRF can be an adjunctive to manage PIs. More future studies are required to confirm the findings. Further studies in the form of randomized trials evaluating the feasibility and cost-effectiveness over a long-term period in spinal cord injury patients with changes in histopathology and growth factor estimation along with platelet concentration are required. To our knowledge till date, no other study in a rehabilitation setting has been done on patients having non-healing ulcers due to varying etiologies over different anatomical locations. It is well established that PIs are more common in persons who are bedridden or wheelchair-dependent, and surgical options like muscle flaps and split skin grafts may further cause loss of strength and gait abnormalities. Further increased shear stress due to spasticity and sensory loss may lead to graft failure and recurrence of PIs. Thus, for such patients, effective conservative options are highly beneficial.
